# Dendritic Cell Served as Salvage Therapy for Advanced Hepatocellular Carcinoma Resistant to Tyrosine Kinase or Immune Checkpoint Inhibitors

**DOI:** 10.3390/cancers18091433

**Published:** 2026-04-30

**Authors:** Wei-Chen Lee, Tsung-Han Wu, Chih-Hsien Cheng, Yin Lai, Hao-Chien Hung, Jin-Chiao Lee, Yu-Chao Wang, Chen-Fang Lee, Ting-Jung Wu, Hong-Shiue Chou, Kun-Ming Chan

**Affiliations:** Department of General Surgery, Linkou Chang-Gung Memorial Hospital, Taoyuan 33357, Taiwan; wutsunghan@gmail.com (T.-H.W.); chengcchj@cgmh.org.tw (C.-H.C.); mr1709@cgmh.org.tw (Y.L.); mp0616@cgmh.org.tw (H.-C.H.); jinchiao@cgmh.org.tw (J.-C.L.); awuang726@gmail.com (Y.-C.W.); lee5310@cgmh.org.tw (C.-F.L.); wutj5056@gmail.com (T.-J.W.); chouhs@cgmh.org.tw (H.-S.C.); chankunming@cgmh.org.tw (K.-M.C.)

**Keywords:** dendritic cell, hepatocellular carcinoma, immune checkpoint inhibitor, tyrosine kinase inhibitor, immunotherapy

## Abstract

Background/objectives: Current treatments for advanced hepatocellular carcinoma (HCC) are tyrosine kinase inhibitors (TKI) and immune checkpoint inhibitors (ICI). When HCC is resistant to TKI or ICI, subsequent available treatments are limited. Dendritic cells (DC) can activate antigen-specific cytotoxic T-cells and may be employed as a subsequent treatment when HCC is resistant to TKI or ICI treatments. Methods: Fifty advanced HCC patients with resistance to ICI or TKI treatment were invited to have autologous DC therapy. DCs were propagated from peripheral blood monocytes and pulsed with tumor lysate. All the patients received ≥3 courses of DC intravenously. For the outcome analysis, 17 patients who were resistant to TKI or other traditional treatments were grouped into A, and 33 patients who were resistant to ICI treatment were grouped into B. Results: For group A patients, the median (interquartile) progression-free and overall survivals were 6 (3–16.3) and 19 (8–24) months, respectively. The 1-, 2- and 3-year overall survivals after DC therapy were 58.2%, 21.8%, and 14.6%, respectively. For group B patients, the median (interquartile) progression-free and overall survivals were 5.0 (4–8) and 9 (5–14.5) months, respectively. The 1-, 2- and 3-year overall survivals after DC therapy were 27.6%, 8.6% and 4.3%, respectively. Taking together 50 patients, the objective response rate was 6.0% and disease control rate was 72.0%. Only three patients (6.0%) had grade I-II hepatitis. Conclusions: DC therapy is a safe treatment for advanced HCC patients. DC can serve as a subsequent therapy to extend the patients’ lives when TKI or ICI treatments are ineffective as advanced HCC treatments.

## Simple Summary

When advanced hepatocellular carcinoma is resistant to tyrosine kinase inhibitors (TKI) or immune checkpoint inhibitors (ICI), subsequent available treatments are limited. Dendritic cell therapy was employed as the subsequent treatment for 17 patients with TKI resistance and 33 patients with ICI resistance. Three patients had objective responses, 33 patients had stable diseases, and only three patients had mild adverse effects. Dendritic cell therapy is a safe treatment and can serve as a subsequent therapy to extend patients’ lives when advanced HCC is resistant to TKI or ICI treatments.

## 1. Introduction

Hepatocellular carcinoma (HCC) is the most common primary malignancy in the liver. The choice of therapeutic modalities depends on the stage of HCC. When the tumors are at the early stage, curative treatments such as liver resection, liver transplantation, and radiofrequency ablation (RFA) can be applied, and patients have the opportunity to be cured [1]. If the tumors are already at a late stage, only non-curative treatments are available, such as transarterial chemoembolization (TACE), tyrosine kinase inhibitors (TKI), immune checkpoint inhibitors (ICI), radiotherapy, chemotherapy, etc. Moreover, it is easy for HCC to recur even after being treated with curative modalities at the early stages. Most HCC will progress to the advanced stages eventually [2,3]. Treatments for advanced HCC are outweighed in importance.

Effective treatments for advanced HCC were lacking until sorafenib was approved to treat advanced HCC in 2008 [4]. Under the treatment of sorafenib, the survival of the advanced HCC patients was significantly prolonged, but the tumor objective response rate was low [4,5]. Later on, lenvatinib was approved to treat advanced HCC with non-inferior results to sorafenib [6]. Immune checkpoint inhibitors (ICI) were also applied to treat advanced HCC and were able to yield a higher objective response rate than TKI [7]. Furthermore, a combination of atezolizumab and bevacizumab was applied to treat advanced HCC with promising effects and was approved to treat advanced HCC. The objective response rate was increased to 30%, with 10% complete response [8]. Survival in patients with advanced HCC was significantly prolonged. However, the tumors will become resistant to the treatments after a period of time, unless the tumors respond to the treatment completely [9,10].

Dendritic cells (DC) are the most potent antigen-presenting cells. When DCs are pulsed with tumor antigens, DCs can activate antigen-specific T-cells to proceed with anti-cancer therapy [11,12]. DC has been applied to treat various malignancies with promising effects [13,14,15,16]. When advanced HCCs are resistant to TKI or ICI treatments, DC may be applied as a subsequent treatment for these advanced HCC. Currently, DC immunotherapy is available to treat advanced malignancies by the Health and Welfare Department of Taiwan under a special regulation. In this study, we would like to report on the outcomes of DC immunotherapy for advanced HCC which were resistant to TKI or ICI treatments.

## 2. Materials and Methods

### 2.1. Patients

This is a retrospective study to collect the data on advanced HCC patients who had DC immunotherapy between July 2020 and June 2025. DC immunotherapy was applied because the advanced HCCs were resistant to traditional therapy, TKI, or ICI treatments. Patients’ profiles, previous treatments, tumors’ response to treatment, and patients’ survival were all collected for analysis. The patients were divided into two groups according to the last ineffective treatment prior to DC immunotherapy. Hence, patients with disease progression after TKI treatment or other traditional treatments were in group A, and patients with disease progression after ICI treatment were in group B. This treatment was approved by the Ministry of Health and Welfare, Taiwan, under special regulation. All the patients signed informed consents. This study conformed to the ethical guidelines of the 2000 Declaration of Helsinki and was approved by institutional review board of Chang-Gung Memorial Hospital, LinKou (IRB No.202300279B0, approved on 31 March 2023).

### 2.2. Clinical Diagnosis of HCC

Diagnosis of HCC was done by dynamic computed tomography (CT) or magnetic resonance imaging (MRI) with typical vascular patterns. A typical vascular pattern was defined as contrast uptake during the arterial phase and washout during the venous and late phases. An a-fetoprotein (AFP) level of more than 400 ng/dL was also diagnostic for HCC.

### 2.3. Inclusion Criteria of DC Immunotherapy

The inclusion criteria for patients to accept DC immunotherapy include (1) signing the informed consent, (2) HCC at Barcelona Clinic Liver Cancer Classification (BCLC) late stage B or C, (3) resistance to traditional therapy, TKI, or ICI treatments, which was defined as tumor progression based on mRECIST criteria, (4) age ≥ 18 years old, and (5) laboratory WBC ≥ 3000/μL, lymphocyte count ≥ 12%, platelet ≥ 80,000/μL, international ratio of prothrombin time ≤ 1.4, and AST/ALT level ≤ 5 folds.

### 2.4. Exclusion Criteria of DC Immunotherapy

The main effect of DC is to activate T-cells. The exclusion criteria for patients to have DC immunotherapy include (1) lymphocyte count < 12%, (2) human immunodeficiency virus infection, (3) uncontrolled infection, (4) acute liver failure, (5) Eastern Cooperative Oncology Group score (ECOG) ≥ 3, (6) failure to obtain tumor specimen, (7) not recovered from adverse effects of other treatments, and (8) expected survival time of <3 months when liver function was in Child–Pugh C status.

### 2.5. Clinical Management

Before DC administration, baseline dynamic computed tomography (CT) or magnetic resonance imaging (MRI) was performed. Laboratory tests of liver function, AFP, and count of blood cells (CBC) were also performed. Three courses of DC at 2-week intervals were administered. After DC administration, laboratory tests of liver function and CBC were measured 2 days after each DC administration. CT/MRI was performed one month after the 3rd course of DC to assess tumor response to the treatment. In the follow-up, laboratory tests of liver function, CBC, AFP, and dynamic CT/MRI were performed every 2–3 months. For the patients with the comforts of DC treatment, DCs might be boosted to enhance anti-tumor immunity and achieve the best survival.

### 2.6. Generation of DC and DC Administration

The propagation of DC was proceeded as per our previous description [15]. Briefly, peripheral blood, of 50–60 mL, was obtained for each DC generation. Monocytes were isolated and suspended in a serum-free AIM-V medium (Life Technologies, Graitherburg, MD, USA). The adherent cells were cultured for 7 days in the medium with recombinant granulocyte-macrophage colony-stimulating factor (1000 U/mL; R&D System Inc., Minneopolis, MN, USA) and interleukin (IL)-4 (1000 U/mL; R&D System Inc., Minneopolis, MN, USA). The DCs were pulsed with autologous tumor lysates 2 days before harvesting and maturated by cocktail cytokines. DCs must meet the quality control of the DC preparation, and was administered intravenously in 5–10 min.

### 2.7. Quality Control of DC Preparation

The quality control criteria include negative for bacteria, negative for mycoplasma, endotoxin < 0.25 EU/mL, viability > 75%, and expression of CD40, CD80, CD83, CD86, and HLA-DR > 60%.

### 2.8. Tumor Lysate Preparation

Tumor specimens were obtained by tumor biopsy or local surgical excision. Tumor cells were dispersed into single cell suspensions (2 × 10^6^ cells/mL) and lysed by three cycles of snap freeze–thawing to obtain tumor lysate. Large particles were removed by centrifuge (600 rpm, 5 min). The lysate was preserved in a −20 °C freezer until it was used to pulse DC.

### 2.9. Tumor Response to DC Immunotherapy

CT or MRI imaging study was performed within 2 weeks prior to DC immunotherapy, one month after the 3rd course of DC immunotherapy, and every 2–3 months in the follow-up period. The tumor response to DC immunotherapy was assessed by modified Response Evaluation Criteria in Solid Tumors (mRECIST) by an independent radiologist [17]. Briefly, complete response (CR) was defined as all target visible lesions disappearing, partial response (PR) was an at least 30% decrease from the baseline sum of the longest diameters, stable disease (SD) was no significant change in the size of target lesions, and progressive disease (PD) was a ≥20% increase in the sum of the longest diameters or the appearance of any new lesions.

### 2.10. Biostatistics

This study included the entire cluster of patients who received DC therapy after having HCCs that were resistant to ICI/TKI treatments. All the available data of the patients were collected. The comparisons of categorical variables were determined by Chi-square or Fisher’s exact tests. The significance of the differences between two groups was determined by unpaired Student’s t-test, and a Mann–Whitney rank sum test was ran when the normality test failed. The significance of the differences between repeated measurements was determined by paired Student’s t-test, and a signed-rank test would be run when normality test was failed. Progression-free survival was defined as from the beginning of DC immunotherapy to the date of tumor progression, and overall survival was defined as from the beginning of DC immunotherapy to the patients’ death. The estimated survival rates were calculated by the Kaplan–Meier method with the log-rank test. The statistical analyses were all performed with SigmaPlot 16 software for Windows (SigmaStat Software, Inc., San Jose, CA, USA). Under a = 0.05 and 1 − b = 0.8, *p* < 0.05 was considered statistically significant.

## 3. Results

### 3.1. Patients

**Figure 1:**
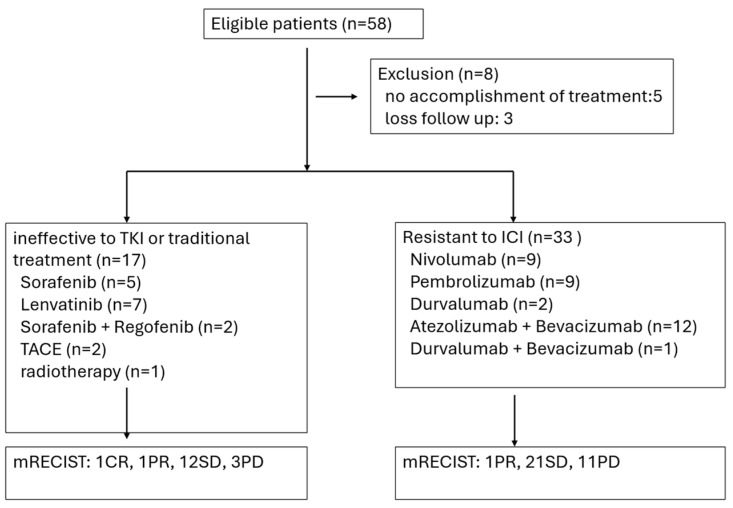
PRISMA flowchart for patients receiving DC immunotherapy.

In total, 50 patients (40 males and 10 females) were included in this study. The patients were divided, with 17 patients in group A and 33 patients in group B (Figure 1). In group A, there were 12 males and five females, and their mean age was 61.9 ± 10.9 years. In group B, there were 28 males and five females, and their mean age was 59.5 ± 13.6 years. Among 50 patients, 35 (70.0%) patients had hepatitis B, seven (14.0%) patients had hepatitis C, three (6.0%) patients had dual hepatitis B and C, and five (10.0%) patients were negative for viral hepatitis. Thirty-one (62.0%) patients had received curative treatments before, but the tumors recurred and progressed to advanced stages. Forty-six of the 50 patients (92.0%) were in BCLC stage C. The clinical profiles of group A and B patients are listed in Table 1.

### 3.2. DC Immunotherapy in Group A Patients

**Figure 2:**
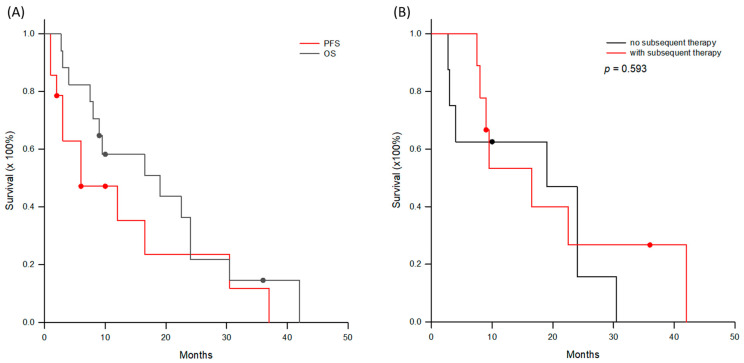
Progression-free and overall survival curves for group A patients. (**A**) The median (interquartile) progression-free and overall survivals were 6 (3–16.5) and 19 (8–24) months, respectively. The 1-, 2-, and 3-year overall survivals were 58.2%, 21.8% and 14.4%, respectively. (**B**) When the patients were further sub-grouped as with (*n* = 9) or without subsequent treatments (*n* = 8), the overall survival was not different (*p* = 0.593).

Seventeen patients in group A began to receive DC immunotherapy from July 2020. All these patients have received traditional HCC treatments before, but the tumors progressed to the advanced stage. At the advanced stage, five patients were treated by sorafenib, seven patients by lenvatinib, and two patients by sequential therapy with sorafenib and regorafenib. The median (interquartile) duration of TKI administration was 8.0 (1–16.5) months, ranging from 0.3 to 52 months. As the patients could not tolerate the side effects of TKI, or the tumors were resistant to TKI treatment and progressed continuously, DC immunotherapy was applied as the subsequent therapy. For these patients, they have already received 2.2 ± 1.0 treatment modalities before DC immunotherapy, such as liver resection, RFA, TACE, radiotherapy, etc. All these patients received three courses of DC immunotherapy, and the median (interquartile) number of DCs administered was 24.3 (10.7–33.3) × 10^6^ cells. Five patients received subsequent additional TAE, and one patient had radiotherapy to induce tumor necrosis for antigen release after the first assessment of tumor response by imaging studies. Three patients were boosted with ICI, and two patients were boosted with an additional three courses of DC immunotherapy. Assessment of tumor response to DC-based immunotherapy showed one CR, one PR, 12 SD, and three PD based on mRECIST criteria. The objective response rate was 11.8% and disease control rate was 82.4%. The median (interquartile) progression-free and overall survivals were 6 (3–16.5) and 19 (8–24) months, respectively. The 1-, 2-, and 3-year overall survivals were 58.2%, 21.8%, and 14.4%, respectively (Figure 2A). When the patients were further sub-grouped as with (*n* = 9) or without subsequent treatments (*n* = 8), overall survival was not different (Figure 2B, *p* = 0.593).

### 3.3. DC Immunotherapy in Group B Patients

**Figure 3:**
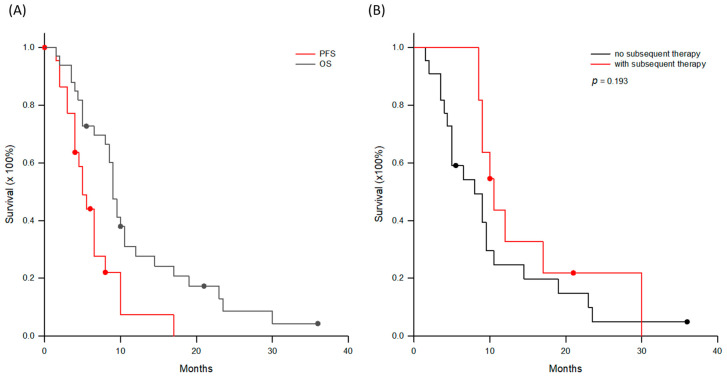
Progression-free and overall survival curves for group B patients. (**A**) The median (interquartile) progression-free and overall survivals were 5.0 (4–8) and 9 (5–14.5) months, respectively. The 1-, 2-, and 3-year overall survivals were 27.6%, 8.6%, and 4.3%, respectively. (**B**) When the patients were further sub-grouped as with (*n* = 11) or without subsequent treatments (*n* = 22), the overall survival was not different (*p* = 0.193).

Thirty-three patients, 28 males and five females, were in group B. All these patients received traditional HCC treatments, including liver resection, RFA, TACE, and radiotherapy therapy. When the tumor progressed to the advanced stage, five patients were treated by sorafenib, eight patients by lenvatinib, and five patients by sequential therapy with sorafenib and regorafenib. The median (interquartile) duration of TKI administration was 6 (2.8–15.3) months and ranged from 1 to 38 months. When the tumors resisted TKI treatment, the treatment was shifted to ICI or ICI was added, including nine nivolumab, nine pembrolizumab, two durvalumab, 12 combinations of atezolizumab and bevacizumab, and one combination of durvalumab and bevacizumab. The median (interquartile) period of ICI administration was 3 (2–5.5) months and ranged from 1.5 to 14 months. When the tumors were resistant to ICI, the patients began to receive DC immunotherapy as the last option of treatment. Thus, these patients had already received 3.2 ± 1.3 treatment modalities before DC immunotherapy. The median (interquartile) interval between the last dose of ICI and DC immunotherapy was 2.8 (2–5) months. The median (interquartile) number of DCs administered was 22.0 (14.3–32.9) × 10^6^ cells. Subsequently, six patients received TAE and three patients received radiotherapy to induce tumor necrosis for tumor antigen release after the first imaging assessment of tumor response. Two patients had additional ICI for boosting. Assessment of tumor response to DC-based immunotherapy showed one PR, 21 SD, and 11 PD based on mRECIST criteria. The objective response rate was 3.0% and disease control rate was 66.7%. The median (interquartile) progression-free and overall survivals were 5.0 (4–8) and 9 (5–14.5) months, respectively. The 1-, 2-, and 3-year overall survivals were 27.6%, 8.6%, and 4.3%, respectively (Figure 3A). When the patients were further sub-grouped as with (*n* = 11) or without subsequent treatments (*n* = 22), the overall survival was not different (Figure 3B, *p* = 0.193).

### 3.4. Outcomes After DC Immunotherapy

**Figure 4:**
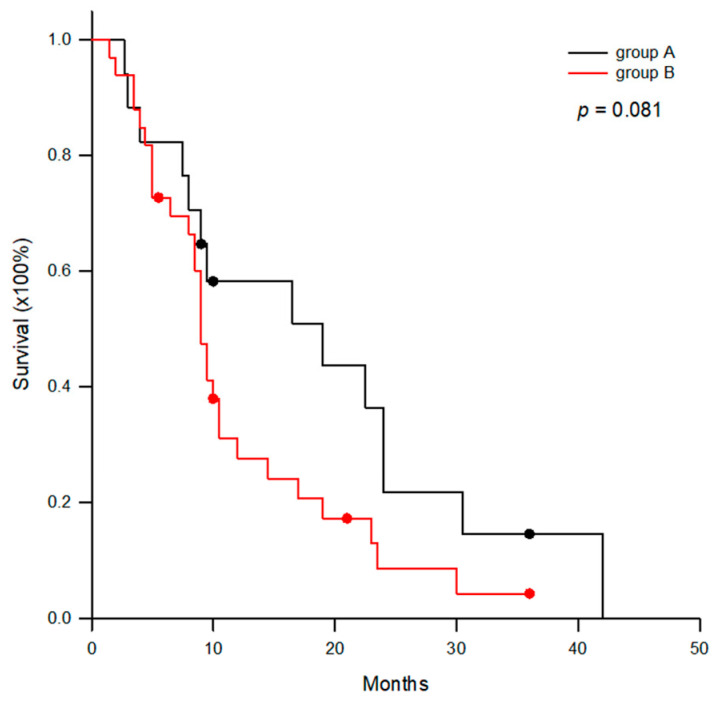
Comparison of overall survival between group A and B patients. The group A patients had a trend of better survival than group B patients when overall survival was calculated with the patients receiving DC immunotherapy (*p* = 0.081).

Regarding therapeutic efficacy, three patients had a partial response to DC immunotherapy and 33 patients had stable diseases in this study, by taking together group A and B patients. The objective response rate was 6.0% and disease control rate was 72%. Group B patients had one more ICI treatment than group A patients prior to DC immunotherapy. Therefore, the group A patients had a trend of better survival than group B patients when overall survival was calculated with the patients receiving DC immunotherapy (Figure 4, *p* = 0.081).

### 3.5. Adverse Effects of DC Immunotherapy

**Figure 5:**
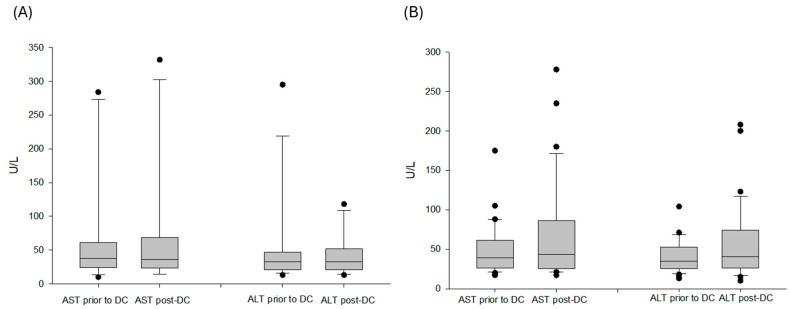
Liver function prior to and after DC immunotherapy in group A and B patients. (**A**) For group A patients, the median (interquartile) level of AST after DC immunotherapy was 36 (23.5–68.5) U/L, compared to 38 (24.5–61) U/L prior to DC immunotherapy (*p* = 0.454). The median (interquartile) level of ALT after DC immunotherapy was 33 (21–52) U/L, compared to 33 (20.5–47) U/L prior to DC immunotherapy (*p* = 0.433). (**B**) For group B patients, the median (interquartile) level of AST after DC immunotherapy was 43 (25.5–86.5) U/L, compared to 39 (25.3–52.8) U/L prior to DC immunotherapy (*p* = 0.224). The median (interquartile) level of ALT after DC immunotherapy was 40.5 (26–73.5) U/L, compared to 34.5 (25.3–52.8) U/L prior to DC immunotherapy (*p* = 0.169).

Among 50 patients, only one (2.0%) patient experienced a mild fever during DC immunotherapy. Concerning autoimmune-like adverse effects, laboratory tests of liver function were measured prior to and after DC immunotherapy. Three patients (6.0%) in group B had grade I-II hepatitis. For group A patients, the median (interquartile) level of AST after DC immunotherapy was 36 (23.5–68.5) U/L, compared to 38 (24.5–61) U/L prior to DC immunotherapy (*p* = 0.454). The median (interquartile) level of ALT after DC immunotherapy was 33 (21–52) U/L, compared to 33 (20.5–47) U/L prior to DC immunotherapy (Figure 5A, *p* = 0.433). For group B patients, the median (interquartile) level of AST after DC immunotherapy was 43 (25.5–86.5) U/L, compared to 39 (25.3–52.8) U/L prior to DC immunotherapy (*p* = 0.224). The median (interquartile) level of ALT after DC immunotherapy was 40.5 (26–73.5) U/L, compared to 34.5 (25.3–52.8) U/L prior to DC immunotherapy (Figure 5B, *p* = 0.169).

### 3.6. A Representative of Partial Response

**Figure 6:**
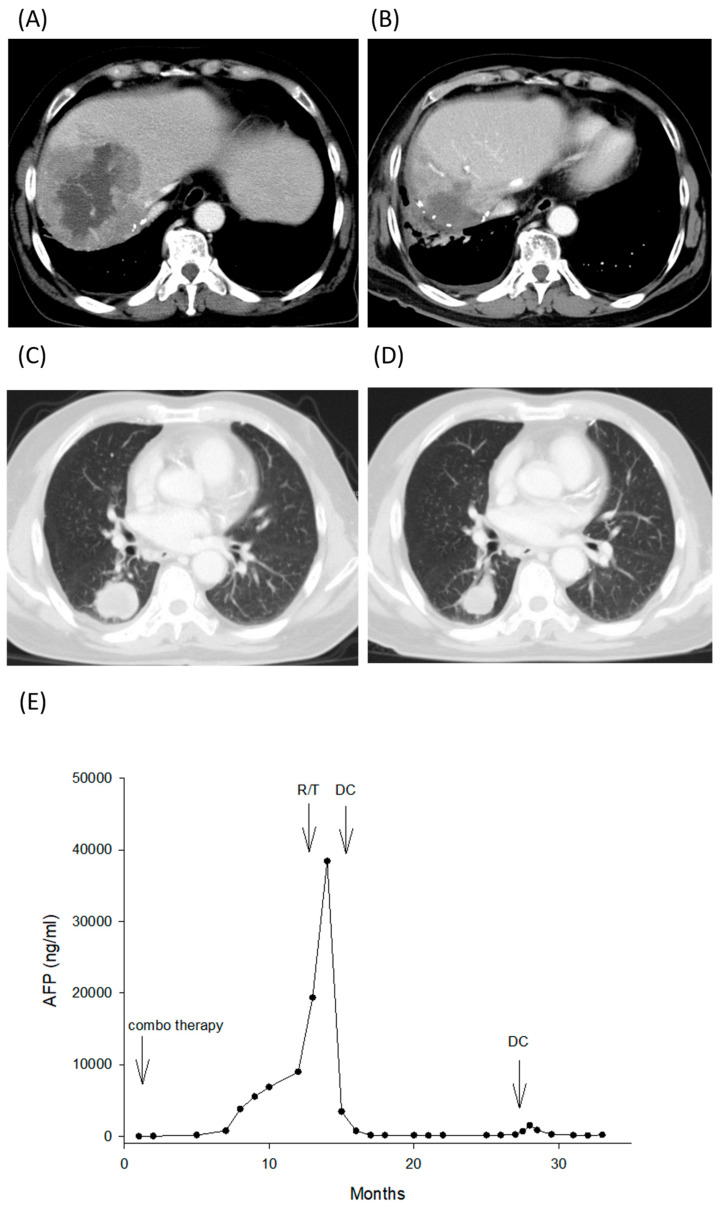
Clinical presentation of a patient with partial response in group B. (**A**) CT showed a 94.1 mm tumor with hepatic vein and inferior vena cava thrombus in the right lobe of the liver. (**B**) The tumor regressed to 44.2 mm in diameter after radiotherapy and DC immunotherapy. (**C**) Lung metastatic tumor began to progress at one year after DC therapy. (**D**) The lung metastatic tumor regressed from 35.1 mm to 24.8 mm after 3 additional courses of DC therapy. (**E**) Serum AFP level was continuously elevated to 3,8452 ng/mL when combination therapy failed to treat the tumors. AFP level decreased to 2242 ng/mL after local radiotherapy. AFP level further decreased to below 200 ng/mL after DC therapy. One year after DC immunotherapy, AFP level was elevated again to 1533 ng/mL when lung tumors progressed. Three courses of DC were boosted. AFP decreased to below 200 ng/mL again.

A hepatitis C patient, aged 60, had a liver resection for HCC in 2013. This patient had a HCC recurrence in 2019, and three times of TACE were performed to control the disease. Later on, the patient joined a clinical trial using a combination of durvalumab and bevacizumab to treat unresectable HCC. After three courses of combination therapy, the patient had ICI-related hepatitis. Prednisolone and mycophenolate mofetil were used to treat the ICI-related adverse effects. Imaging study was performed and showed tumor progression with hepatic vein and inferior vena cava (IVC) thrombus, lung metastasis, and bile duct invasion (Figure 6A). Percutaneous transhepatic cholangiography and drainage were performed to release jaundice. As the tumor progressed quickly, radiotherapy was arranged while awaiting DC preparation. Then, radiotherapy, 4000 cGy, for local disease control was performed and followed by three courses of DC immunotherapy. Radiotherapy had a therapeutic effect, decreasing AFP level from 3,8452 ng/mL to 2242 ng/mL. After DC immunotherapy, AFP further decreased to below 200 ng/mL. CT showed disappearance of hepatic vein and IVC thrombus and tumor regression, from 94.1 to 44.2 mm in diameter (Figure 6B). One year after DC immunotherapy, AFP elevated again to 1533 ng/mL and lung tumors progressed (Figure 6C). Three courses of DC were boosted. AFP decreased to below 200 ng/mL again and the imaging study showed that lung metastatic tumor regressed from 35.1 mm to 24.8 mm (Figure 6D). In December 2022, this patient got pneumonia and further developed a pleurobiliary fistula. The patient became sick and HCC progressed again. The patient died of pneumonia in March 2023. Progression-free survival for this patient was 17 months, and survival time was 23.5 months since he received DC immunotherapy.

## 4. Discussion

Advanced HCC is a highly malignant disease with very poor outcomes. Before sorafenib was introduced to treat advanced HCC, there was no effective treatment for advanced HCC, and the lifespans of patients were only several months. When sorafenib was applied to treat advanced HCC patients, the median overall survival was significantly prolonged from 4.2 to 7.9 months to 6.5–10.7 months [4,5]. Although advanced HCC became treatable, the prognosis was still poor. Later on, ICI was applied to treat advanced HCC and yielded promising effects [7]. Recently, a combination of ICI and TKI became the common regimen of treatment, and objective response rate was increased to 25–30% [8,18]. The patients’ survivals were significantly prolonged if they had an objective response to the treatments. However, when the tumors are resistant to the TKI/ICI treatments and progress again, the treatments then become a struggle. It is essential to find a subsequent treatment to treat these patients safely.

DC is the most potent antigen-presenting cell and can activate antigen-specific cytotoxic T-cells. Theoretically, cancer cells are developed from cell transformation due to enhancement of oncogenes or loss of suppressor genes. The genetic alteration leads transformed cancer cells to express tumor-specific or tumor-associated antigens. DCs can pick up these antigens, process the antigens, and express the antigens to provoke antigen-specific T-cells. These T-cells will execute antigen-specific cytotoxicity to eradicate cancer cells [11]. Under such a mechanism, DC has been applied to treat various cancers with promising effects [13,15,19,20]. DC has also been used to treat HCC [16]. Hence, we applied DCs to treat advanced HCC when the HCCs were resistant to TKI/ICI treatments in this study.

DCs were still effective in treating the advanced HCCs, which were already resistant to TKI and other traditional treatments. The therapeutic results showed that DC immunotherapy could yield 6 months of median progression-free survival and 19 months of median overall survival. Sorafenib was the first TKI used to treat advanced HCC and launched medical treatment for HCC. Survival of the advanced HCC patients could be prolonged for several months [4]. Lenvatinib was also introduced to treat advanced HCC after the clinical trial approved that its therapeutic effect was not inferior to sorafenib [6]. Regorafenib was the second-line TKI used to conduct sequential therapy for HCC after sorafenib failed to treat the disease [21]. Although TKI has been a cornerstone of advanced HCC treatment, acquired resistance to TKI develops during treatment. The mechanisms of developing TKI resistance are diverse and complex, including target alteration, aberrant activation of signaling pathways, and influence of tumor microenvironment. When these TKIs are no longer effective to treat the tumors, ICI can be applied as the second-line treatment. The alternative treatment is DC immunotherapy. Our colleagues have reported the therapeutic results of 120 patients who were resistant to sorafenib or lenvatinib treatment and received nivolumab or pembrolizumab as the second-line treatment. The median progression-free survival was 2.7 and 2.9 months, and median overall survival was 10.8 months and 8.1 months for nivolumab and pembrolizumab, respectively [22]. DCs pulsed with tumor-associated antigens can activate antigen-specific cytotoxic T-cells to target cancer cells, which bypasses the mechanisms of TKI resistance. Therefore, DC immunotherapy is still effective in treating advanced HCC, which is resistant to TKI. Although ICI has almost become a standard treatment for advanced HCC, DC immunotherapy in this study could yield non-inferior therapeutic outcomes to ICI, or even better. Under such difficulties in treatment, application of DC immunotherapy as a subsequent therapy could achieve 54.5% 1-year survival and 18.2% 2-year survival.

ICI was introduced to treat advanced HCC since 2017, when nivolumab was used to treat advanced HCC and showed therapeutic effects [7]. Recently, a combination of atezolizumab and bevacizumab was approved to treat advanced HCC and yielded around a 30% objective response rate and became the first-line treatment for advanced HCC [8]. Other ICIs were also introduced to treat advanced HCC with a certain degree of objective response [23,24,25]. ICI can reactivate exhausted T-lymphocytes to target cancer cells. However, advanced HCC may be resistant to ICI treatment due to defective antigen presentation, alternative checkpoint pathways, tumor microenvironment, or metabolic effects. Considering defective antigen presentation, ex vivo propagated DCs pulsed with tumor lysates may compensate for antigen presentation and activate antigen-specific T-cells to proceed specific cancer cell eradication. In current practices, next-line treatments are limited when the tumors are resistant to the ICI treatment. In this study, autologous DC immunotherapy was applied to treat advanced HCC after the tumors were resistant to ICI treatment. For all these patients, DC immunotherapy can still yield 5 months of progression-free survival and 9 months of overall survival. One patient even had partial response to DC immunotherapy. These therapeutic results were similar to those of durvalumab re-challenge in advanced hepatocellular carcinoma refractory to prior anti-PD-1 therapy reported by our colleagues [26]. DC immunotherapy can be applied as a next-line treatment for advanced HCC which was resistant to ICI treatment.

DC therapy is basically an immunotherapy, and the therapeutic effects depend on the hosts’ immunity. For the patients in group B, they are already resistant to TKI and ICI treatments, and their immunities are supposed to be exhausted and low. Survival after DC immunotherapy had an inferior trend to that of group A patients. However, if survival was calculated from the beginning of the ICI treatment for group B patients, the survival was similar to that of group A patients. In this study, five patients in group A and six patients in group B had subsequent TAE therapy. One patient in group A and three patients in group B had subsequent radiotherapy. TAE is not recommended for advanced HCC in standard practice. TAE in this study was to induce tumor necrosis for the releasing of tumor antigens to reactive memory-like T-cells which were provoked by DC. In our previous animal study, tumor antigens themselves could further reactive memory T-cells to increase therapeutic effects after DC therapy [27]. However, improvement in survival was not seen in the patients with subsequent TAE or radiotherapy.

A treatment with low adverse effects and without discomfort is important for late-stage cancer patients [28]. DC immunotherapy is a safe treatment with low adverse effects for advanced HCC patients. In this study, the hepatocellular cancer cell lysate was used as tumor antigens to pulse DC. Autoimmune-like hepatitis was of the most concern for this DC immunotherapy. Among 50 patients, only one patient experienced mild fever after DC infusion, and three patients had grade I to II hepatitis after DC immunotherapy. The hepatitis recovered spontaneously. Hence, DC immunotherapy is a safe treatment for advanced HCC patients.

Treatments are difficult when HCCs are already resistant to TKI/ICI. Taking group A and B patients together, the objective response rate was only 6.0%, but the disease control rate could reach 72.0%. Thereafter, the overall survival could be prolonged by DC therapy. However, the treatment results of DC therapy are not satisfactory. There are many possible mechanisms to impair DC treatment for cancers such as loss of tumor antigen expression and lack of co-stimulation. Tumor cells may inhibit DC by releasing a-catenin, reactive oxygen species, VEGF, IL-10, PGE2, TGF-b, etc. Regulatory T-cells and myeloid-derived suppressor cells may be induced in tumor microenvironments and they may interfere with the antigen presentation of DC to T-cells [29,30]. The immunosuppressive entity of the tumor microenvironment itself may impede the function of DC-induced cytotoxic T-cells. All these mechanisms may let tumors escape anti-tumor immunity and result in treatment failures. Further studies are needed to improve the therapeutic effects of DC.

## 5. Conclusions

Current popular treatments for advanced HCC are TKI and ICI. When the tumors are resistant to TKI/ICI, the next available treatments are limited and patients’ survival is poor. DC immunotherapy can be applied as a therapeutic option for advanced HCC to prolong patients’ survival. The therapeutic results in this study are encouraged. However, large-scale randomized control trials to compare other second-line treatments are needed to validate the therapeutic effects of DC therapy.

## Figures and Tables

**Table 1:** The clinical profiles of group A and B patients with DC immunotherapy.

Patients	Group A (*n* = 17)	Group B (*n* = 33)	*p*
Gender (M/F)	12/5	28/5	0.277
Age (years)	61.9 ± 10.9	59.5 ± 13.6	0.516
Hepatitis B( + ) C(−) B(−) C( + ) B( + ) C( + ) B(−) C(−)	11 2 3 1	24 5 0 2	0.091
Previous treatment Hepatectomy RFA TACE (TARE) Radiotherapy Proton therapy TKI ICI	8 1 12 5 1 16 0	18 5 18 11 2 24 33	0.005
BCLC stage B C	1 16	3 30	1.000
Response (mRECIST) CR PR SD P	1 1 12 3	0 1 21 11	0.356
Subsequent treatment TAE Radiotherapy ICI	5 1 3	6 3 2 (pembrolizumab)	0.390

M, male; F, female; B, hepatitis B; C, hepatitis C; RFA, radiofrequency; TACE, transcatheter arterial chemoembolization; TARE, transcatheter arterial radioembolization; TKI, tyrosine kinase inhibitor; ICI, immune checkpoint inhibitor; BCLC, Barcelona Clinic Liver Cancer Classification; CR, complete response; PR, partial response; SD, stable disease; PD, progressive disease; N/A, non-assessment; TAE, transcatheter arterial embolization.

## Data Availability

The authors confirm that the data supporting the findings of this study are available within the article.

## Abbreviations

The following abbreviations are used in this manuscript:

AFP	a-fetoprotein
ALT	Alanine aminotransferase
AST	Aspartate aminotransferase
BCLC	Barcelona Clinic Liver Cancer Classification
CT	Computed tomography
DC	Dendritic cell
HCC	Hepatocellular carcinoma
ICI	Immune checkpoint inhibitor
MRI	Magnetic resonance imaging
PR	Partial response
SD	Stable disease
PD	Progressive disease
mRECIST	Modified response evaluation criteria in solid tumors
TKI	Tyrosine kinase inhibitor

## References

[1] Reig M., Forner A., Rimola J., Ferrer-Fabrega J., Burrel M., Garcia-Criado A., Kelley R.K., Galle P.R., Mazzaferro V., Salem R. (2022). BCLC strategy for prognosis prediction and treatment recommendation: The 2022 update. J. Hepatol..

[2] Lee W.C., Lee C.F., Cheng C.H., Wu T.J., Chou H.S., Wu T.H., Soong R.S., Chan K.M., Yu M.C., Chen M.F. (2015). Outcomes of liver resection for hepatocellular carcinoma in liver transplantation era. Eur. J. Surg. Oncol..

[3] Imamura H., Matsuyama Y., Tanaka E., Ohkubo T., Hasegawa K., Miyagawa S., Sugawara Y., Minagawa M., Takayama T., Kawasaki S. (2003). Risk factors contributing to early and late phase intrahepatic recurrence of hepatocellular carcinoma after hepatectomy. J. Hepatol..

[4] Llovet J.M., Ricci S., Mazzaferro V., Hilgard P., Gane E., Blanc J.-F., de Oliveira A.C., Santoro A., Raoul J.-L., Forner A. (2008). Sorafenib in Advanced Hepatocellular Carcinoma. N. Engl. J. Med..

[5] Cheng A.-L., Kang Y.-K., Chen Z., Tsao C.-J., Qin S., Kim J.S., Luo R., Feng J., Ye S., Yang T.-S. (2009). Efficacy and safety of sorafenib in patients in the Asia-Pacifi c region with advanced hepatocellular carcinoma: A phase III randomised, double-blind, placebo-controlled trial. Lancet Oncol..

[6] Kudo M., Finn R.S., Qin S., Han K.-H., Ikeda K., Piscaglia F., Baron A., Park J.-W., Han G., Jassem J. (2018). Lenvatinib versus sorafenib in first-line treatment of patients with unresectable hepatocellular carcinoma: A randomised phase 3 non-inferiority trial. Lancet.

[7] El-Khoueiry A.B., Sangro B., Yau T., Crocenzi T.S., Kudo M., Hsu C., Kim T.-Y., Choo S.-P., Trojan J., Welling T.H. (2017). Nivolumab in patients with advanced hepatocellular carcinoma (CheckMate 040): An open-label, non-comparative, phase 1/2 dose escalation and expansion trial. Lancet.

[8] Finn R.S., Qin S., Ikeda M., Galle P.R., Ducreux M., Kim T.Y., Kudo M., Breder V., Merle P., Kaseb A.O. (2020). Atezolizumab plus Bevacizumab in Unresectable Hepatocellular Carcinoma. N. Engl. J. Med..

[9] Jenkins R.W., Barbie D.A., Flaherty K.T. (2018). Mechanisms of resistance to immune checkpoint inhibitors. Br. J. Cancer.

[10] Weiss S.A., Sznol M. (2021). Resistance mechanisms to checkpoint inhibitors. Curr. Opin. Immunol..

[11] Santos P.M., Butterfield L.H. (2018). Dendritic Cell-Based Cancer Vaccines. J. Immunol..

[12] Girolomoni G., Ricciardi-Castagnoli P. (1997). Dendritic cells hold promise for immunotherapy. Immunol. Today.

[13] Fong L., Engleman E.G. (2000). Dendritic cells in cancer immunotherapy. Annu. Rev. Immunol..

[14] Bol K.F., Schreibelt G., Gerritsen W.R., de Vries I.J., Figdor C.G. (2016). Dendritic Cell-Based Immunotherapy: State of the Art and Beyond. Clin. Cancer Res..

[15] Lee W.-C., Wang H.-C., Hung C.-F., Huang P.-F., Lia C.-R., Chen M.-F. (2005). Vaccination of Advanced Hepatocellular Carcinoma Patients with Tumor Lysate-Pulsed Dendritic Cells-A Clinical Trial. J. Immunother..

[16] Chen C., Ma Y.H., Zhang Y.T., Zhang F., Zhou N., Wang X., Liu T., Li Y.M. (2018). Effect of dendritic cell-based immunotherapy on hepatocellular carcinoma: A systematic review and meta-analysis. Cytotherapy.

[17] Lencioni R., Llovet J.M. (2010). Modified RECIST (mRECIST) assessment for hepatocellular carcinoma. Semin. Liver Dis..

[18] Raybould A.L., Sanoff H. (2020). Combination Antiangiogenic and Immunotherapy for Advanced Hepatocellular Carcinoma: Evidence to Date. J. Hepatocell. Carcinoma.

[19] Draube A., Klein-Gonzalez N., Mattheus S., Brillant C., Hellmich M., Engert A., von Bergwelt-Baildon M. (2011). Dendritic cell based tumor vaccination in prostate and renal cell cancer: A systematic review and meta-analysis. PLoS ONE.

[20] Lee J.H., Tak W.Y., Lee Y., Heo M.K., Song J.S., Kim H.Y., Park S.Y., Bae S.H., Lee J.H., Heo J. (2017). Adjuvant immunotherapy with autologous dendritic cells for hepatocellular carcinoma, randomized phase II study. Oncoimmunology.

[21] Bruix J., Qin S., Merle P., Granito A., Huang Y.-H., Bodoky G., Pracht M., Yokosuka O., Rosmorduc O., Breder V. (2017). Regorafenib for patients with hepatocellular carcinoma who progressed on sorafenib treatment (RESORCE): A randomised, double-blind, placebo-controlled, phase 3 trial. Lancet.

[22] Chen Y.H., Tsai C.H., Chen Y.Y., Wang C.C., Wang J.H., Hung C.H., Kuo Y.H. (2023). Real-world comparison of pembrolizumab and nivolumab in advanced hepatocellular carcinoma. BMC Cancer.

[23] Vaddepally R.K., Kharel P., Pandey R., Garje R., Chandra A.B. (2020). Review of Indications of FDA-Approved Immune Checkpoint Inhibitors per NCCN Guidelines with the Level of Evidence. Cancers.

[24] Pinter M., Jain R.K., Duda D.G. (2021). The Current Landscape of Immune Checkpoint Blockade in Hepatocellular Carcinoma: A Review. JAMA Oncol..

[25] Giraud J., Chalopin D., Blanc J.F., Saleh M. (2021). Hepatocellular Carcinoma Immune Landscape and the Potential of Immunotherapies. Front. Immunol..

[26] Lai K.C., Chen Y.H., Hung Y.P., Chiang N.J., Chen M.H., Chen S.C. (2024). Efficacy and safety of durvalumab rechallenge in advanced hepatocellular carcinoma patients refractory to prior anti-PD-1 therapy. Hepatol. Int..

[27] Lee W.C., Cheng C.H., Lee C.F., Hsu H.Y., Hsu P.Y., Wu T.J., Chan K.M. (2022). Enhancement of dendritic cell immunotherapy by recalling antigens for hepatocellular carcinoma in mice. Immunotherapy.

[28] Anguille S., Smits E.L., Lion E., van Tendeloo V.F., Berneman Z.N. (2014). Clinical use of dendritic cells for cancer therapy. Lancet Oncol..

[29] Sadeghzadeh M., Bornehdeli S., Mohahammadrezakhani H., Abolghasemi M., Poursaei E., Asadi M., Zafari V., Aghebati-Maleki L., Shanehbandi D. (2020). Dendritic cell therapy in cancer treatment; the state-of-the-art. Life Sci..

[30] Mestrallet G., Sone K., Bhardwaj N. (2022). Strategies to overcome DC dysregulation in the tumor microenvironment. Front. Immunol..

